# Smoking increases the risk of post-acute COVID-19 syndrome: Results from a French community-based survey

**DOI:** 10.18332/tid/150295

**Published:** 2022-06-17

**Authors:** Hugues Barthélémy, Emmanuelle Mougenot, Martin Duracinsky, Dominique Salmon-Ceron, Jennifer Bonini, Fabienne Péretz, Olivier Chassany, Patrizia Carrieri

**Affiliations:** 1 Unité de Recherche Clinique, Centre Hospitalier d’Auxerre, Auxerre, France; 2 Patient-Centered Reported Outcomes, Université de Paris, Paris, France; 3 Unité de Recherche Clinique en Economie de la Santé, Assistance Publique - Hôpitaux de Paris, Paris, France; 4 Service Maladies Infectieuses et Tropicales, Assistance Publique - Hôpitaux de Paris, Paris, France; 5 Université Paris Descartes, Paris, France; 6 Abelia Science, Saint-Georges-sur-Baulche, France; 7 Aix Marseille Univ, Inserm, IRD, SESSTIM, Sciences Economiques & Sociales de la Santé & Traitement de l’Information Médicale, ISSPAM, Marseille, France

**Keywords:** France, post-acute COVID-19 syndrome, social media, surveys and questionnaires, smoking

## Abstract

**INTRODUCTION:**

We aimed to estimate the prevalence and incidence of specific symptoms and predictors of post-acute COVID-19 syndrome using data collected from an anonymous online survey.

**METHODS:**

We included adult participants with symptoms ≥60 days (D60+), fulfilling the World Health Organization COVID-19 cases definition, and/or hospitalized for COVID-19 at the time of infection (D0). Self-reported symptoms were collected at D0 and D60+. Logistic regression was performed to identify factors associated with self-reported cutaneous signs prevalence and self-reported tachycardia and/ or HBP incidence on D60+.

**RESULTS:**

From April to June 2020, 956 members of a Twitter long-term COVID-19 community were included in the study population: 81% were women, 81% were aged <50 year, 22% were smokers, and 95% have never been hospitalized. At D60+, the 956 participants reported a broad spectrum of symptoms which were also present at D0+. At D60+, 16% and 39% of participants reported cutaneous signs and tachycardia and/or hypertension, respectively. The incidence of self-reported tachycardia and/or hypertension at D60+ was 12%. Female gender (AOR=2.56; 95% CI: 1.22–6.1) and smoking (AOR=2.34; 95% CI: 1.39–3.92) were associated with prevalence of cutaneous signs at D60+. Smoking (AOR=2.05; 95% CI: 1.2– 3.47) was the main correlate of tachycardia and/or HBP incidence at D60+.

**CONCLUSIONS:**

The incidence of self-reported tachycardia and/or hypertension is not negligible and suggests an interaction between COVID-19 and smoking. Reinforcing symptoms monitoring of people after acute COVID-19, mainly women and smokers, and expanding the promotion of smoking cessation strategies are novel priorities in this COVID-19 era.

## INTRODUCTION

In mid December 2019, a new coronavirus responsible for COVID-19 appeared, causing a global pandemic^1^. To prevent health systems being rapidly overwhelmed, many countries around the world implemented restrictions on people’s movement to slow the spread of the SARS-CoV-2 virus^2^. This unprecedented situation and the lack of knowledge about this new disease gave rise to questions and concerns for patients, caregivers, and the scientific community. Later, the testimonies of individuals reporting suffering from long-term symptoms after SARS-CoV-2 infection accumulated. These observations were the subject of a World Health Organization (WHO) presentation mentioning that, for some people, some symptoms may persist or recur for weeks or months following initial recovery. This can also happen in people with mild disease^3^. Since then, the number of scientific publications focusing on long-term COVID-19 symptoms has increased from <20 in 2020 to >300 in 2021. It is currently recognized that post-acute COVID-19 syndrome, also referenced by terms such as ‘post-COVID syndrome’, ‘long COVID’, ‘persistent long COVID’, or ‘post-acute COVID sequelae (PACS)’, is a pathological condition which involves persistent physical, medical and cognitive sequelae following acute COVID-19^4^. Like COVID-19, post-acute COVID-19 syndrome can affect many systems and multiple organs, including respiratory, cardiovascular, neurological, gastrointestinal and musculoskeletal systems^5,6^. There is clear evidence that post-acute COVID-19 syndrome can affect the whole spectrum of COVID-19 patients^5^, and that about 20% and 10% of SARS-CoV-2 positive patients have symptoms persisting for at least 5 or 12 weeks, respectively^7^.

During the pandemic, Twitter was an effective means of knowing how populations reacted to the COVID-19 health crisis^8^, including tracking mental health^9^ and identifying a wider range of COVID-19 symptoms^10,11^ besides the most common symptoms of acute infection (cough, fever, asthenia, myalgia, and impaired smell and taste including anosmia and ageusia)^12^.

In France, during the first lockdown period, marked by worry and fear of the unknown, people infected by the SARS-CoV-2 virus with non-severe symptoms were advised to stay at home. During this period, a community called *#aprèsJ20* (‘After Day 20’) was created on Twitter, bringing together individuals who claimed to have experienced COVID-19 and to present or have presented symptoms more than 20 days after SARS-CoV-2 infection. In April 2020, a member of this community launched an anonymous online survey so that people could share their experiences and support and reassure each other.

This study aimed to describe long-term self-reported symptoms in this community and identify correlates of less explored but frequent long-term symptoms after COVID-19, such as cutaneous signs (such as itching, red patches or pseudo-chilblain lesions) and incident tachycardia and/or high blood pressure (HBP). Indeed, these two symptoms were considered relevant by the member of the community who launched the study although less frequently investigated than fatigue or ageusia/anosmia in the literature^6,13-15^.

## METHODS

A member of the *#aprèsJ20* Twitter community launched an anonymous online survey using a Google Form (no IP addresses collected). People who agreed to participate clicked on a shared link and were free to move forward in the survey by clicking as they wished. They knew that their answers would be collected, gathered, analyzed, and visible to everyone.

All individuals who completed at least one item of the survey were eligible for the study. Only adult participants living in France, fulfilling the World Health Organization COVID-19 cases definition, and/or hospitalized for acute COVID-19 at the time of infection (D0), and reporting symptoms at least 60 days after D0 (i.e. D60+), were included in the study. WHO COVID-19 definition is based on either laboratory confirmation of SARS-CoV-2 infection (Polymerase Chain Reaction -PCR- or serology), chest imaging showing findings suggestive of COVID-19, sudden anosmia and ageusia in the absence of any other identified cause, or a combination of evocative symptoms (i.e. acute onset of fever and cough, or of any three or more of the following signs or symptoms: fever, cough, general weakness/fatigue, headache, myalgia, sore throat, coryza, dyspnea, anorexia/ nausea/vomiting, diarrhea, and altered mental status)^16^.

The survey included items (closed or open questions) about location, sociodemographic characteristics, smoking habits, blood group, oxygen level, SARS-CoV-2 PCR, serology, chest imaging, medical management and follow-up, feelings, as well as symptoms experienced either at D0 or later. Due to the design of the survey (‘After Day 20’), symptoms were always collected retrospectively at D0.

### Statistical analysis

Statistical analyses were performed on R software, version 3.6.3 (R Core team). Only answers to closed questions were analyzed. A descriptive analysis of the participants’ characteristics and symptoms at D0 and D60+ was conducted on the study population. The prevalence on D0 and D60+, and the persistence (i.e. presence at D0 and D60+), and incidence at D60+ (i.e. absence at D0 and presence at D60+) were calculated for each symptom. Univariate and multivariate logistic regression analyses were carried out: 1) to compare participants with and without prevalent self-reported cutaneous signs at D60+ and identify baseline factors associated with the prevalence of self-reported cutaneous signs, and 2) to compare participants with and without incident self-reported tachycardia and/or HBP at D60+ and identify baseline factors associated with the incidence of self-reported tachycardia and/ or HBP at D60+. Covariates with a liberal p≤0.2 were considered eligible in the multivariate logistic regression model and maintained if significantly (p<0.05) associated with outcome. Crude odds ratios (univariate analysis, ORs) and adjusted odds ratios (multivariate analysis, AORs) are reported with 95% confidence intervals (CI).

As this study used completely anonymous data from voluntary participants, no oral or written consent was required. The use of the database was approved by an independent French review board CERF, Hôpital Foch, Paris, France. No monetary incentive was offered to participants, who could access a real-time update on survey results at any time by simply clicking on an active link.

## RESULTS

### Study population

**Figure 1:**
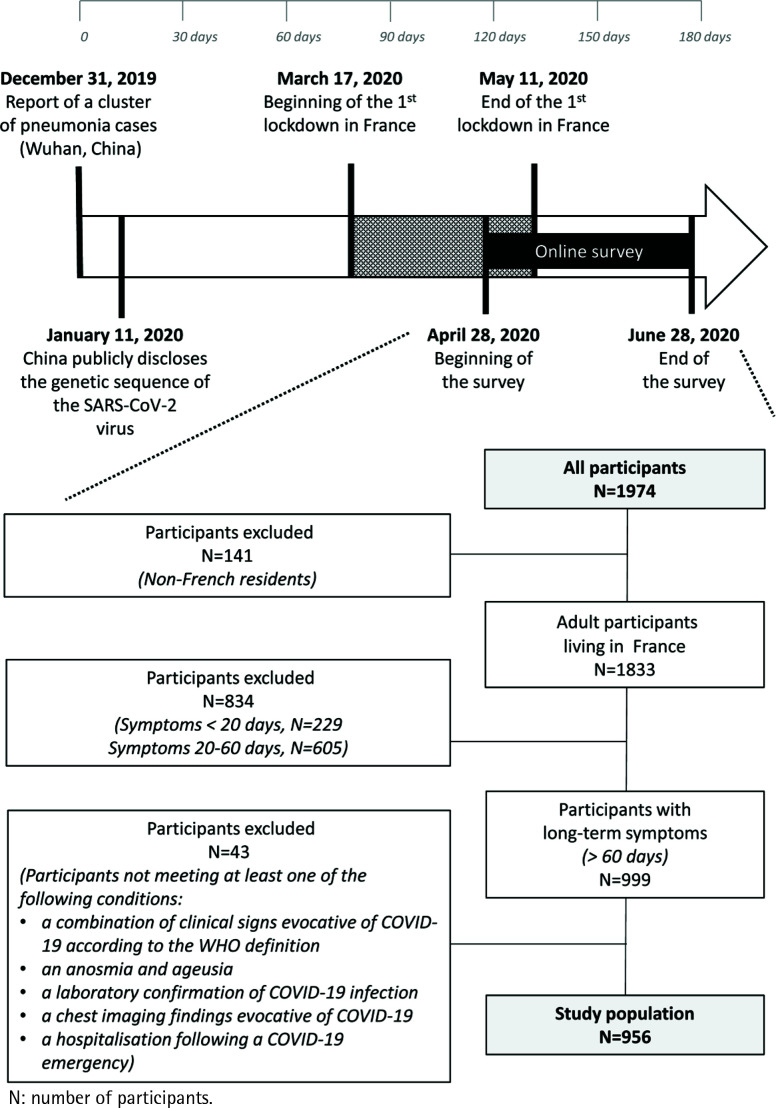
Study flowchart (time and participants)

From 28 April to 28 June 2020, 1974 members of the Twitter community participated in the online survey and 956 who fulfilled the inclusion criteria constituted the study population (Figure 1).

The 956 participants were distributed throughout France (Supplementary file Figure 1); 81% were female, 81% were aged ≤50 years, and 22% were smokers (e-cigarette included). At D0, 377 participants attended an emergency department, and of these, 43 (11%) were hospitalized. Overall, 95% of the participants reported not having been hospitalized, 49% not to have been medically monitored, and 81% claimed to feel abandoned (Table 1).

**Table 1:** Characteristics of the study population (N=956)

*Characteristics*	*MD*	*n*	*%*
**Gender**	0	956	
Male		181	19
Female		775	81
**Age** (years)	2	954	
20–40		404	42
40–50		368	39
>50		182	19
**Smoking**	19	937	
Yes		210	22
No		727	78
**Blood group**	71	885	
O		364	41
Other		521	59
**Lowest oxygen saturation level**	351	605	
<95%		207	34
**SARS-CoV-2 test**	264	692	
Tested (biological test)		230	33
PCR+ (alone)		91	13
Serology+ (alone)		91	13
PCR+ and serology+		48	7
Not tested		462	67
**Chest imaging evocative of COVID-19**	0	956	
Yes		163	17
**Attending emergency department**	7	949	
Yes		377	40
Hospitalized during the visit		43	5
No		572	60
**Resuscitation**	20	936	
Yes		7	1
No		929	99
**One or more treatments administered for COVID-19**	29	927	
Yes		516	56
No		411	44
**Medically monitored for the COVID-19**	66	890	
Yes		453	51
No		437	49
**Feelings of medical abandonment**	19	937	
Yes		755	81
No		182	19

MD: number of missing data. PCR: polymerase chain reaction.

### Prevalence of symptoms at D0 and D60+

**Figure 2:**
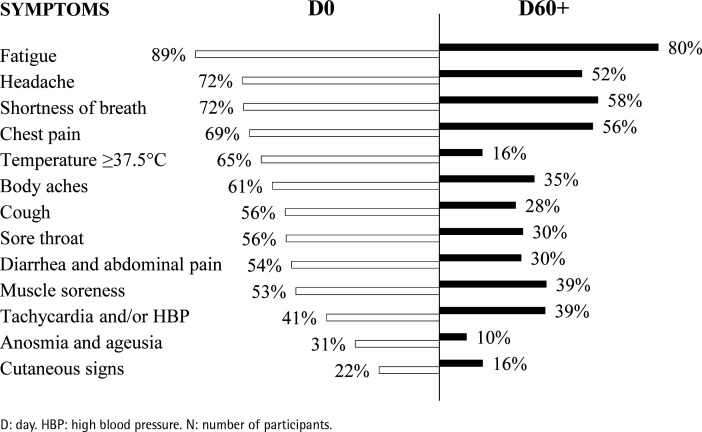
Self-reported symptoms during COVID-19 (D0) and more than 60 days later (D60+) (N=956)

At D0, the most prevalent self-reported symptoms were fatigue (89%), headache (72%), shortness of breath (72%), chest pain (69%), body temperature ≥37.5°C (65%), and body aches (61%). Tachycardia and/or HBP, anosmia and ageusia, and cutaneous signs were the less prevalent self-reported symptoms (41%, 31%, and 21%, respectively) (Figure 2).

At D60+, the most prevalent self-reported symptoms were fatigue (80%), shortness of breath (58%), chest pain (56%), and headache (52%), followed by tachycardia and/or HBP (39%) and muscle soreness (39%). Cutaneous signs (16%), fever (body temperature ≥37.5°C) (16%), and anosmia and ageusia (10%) were the less prevalent self-reported symptoms at D60+ (Figure 2).

### Persistence or incidence of symptoms at D60+

Most of self-reported symptoms at D60+ were persistent, being also reported by the patients at D0, e.g. 97%, 93%, and 89% of participants who reported fever, fatigue, or anosmia and ageusia at D60+ reported having the symptom at D0 (Table 2). Few self-reported symptoms at D60+ occurred after D0, except for self-reported tachycardia and/ or hypertension which were reported by 12% of participants at D60+ but not at D0. On the other hand, anosmia and ageusia were the less frequently reported incident symptoms, being incident in 1% of the participants at D60+ (Table 2).

**Table 2:** Self-reported symptoms during COVID-19 (D0) and 60 days or more later (D60+) in the study population (N=956)

*Symptoms*	*D0*				*D60+*				*D60+: Presence*							
	*Absence*		*Presence*		*Absence*		*Presence*		*D0: Presence*				*D0: Absence*			
	*N1*	*N1/N %*	*N2*	*N2/N %^a^*	*N3*	*N3/N %*	*N4*	*N4/N %^b^*	*N5*	*N5/N %^c^*	*N5/N2 %*	*N5/N4 %*	*N6*	*N6/N %^d^*	*N6/N1 %*	*N6/N4 %*
Fatigue	104	11	852	89	192	20	764	80	714	75	84	93	50	5	48	7
Shortness of breath	271	28	685	72	403	42	553	58	481	50	70	87	72	8	27	13
Headache	268	28	688	72	459	48	497	52	439	46	64	88	58	6	22	12
Chest pain	292	31	664	69	421	44	535	56	449	47	68	84	86	9	29	16
Body temperature ≥ 37.5 °C	333	35	623	65	807	84	149	16	144	15	23	97	5	1	2	3
Body aches	372	39	584	61	625	65	331	35	271	28	46	82	60	6	16	18
Cough	417	44	539	56	690	72	266	28	235	25	44	88	31	3	7	12
Sore throat	420	44	536	56	665	70	291	30	241	25	45	83	50	5	12	17
Diarrhea and abdominal pain	439	46	517	54	671	70	285	30	231	24	45	81	54	6	12	19
Muscle soreness	454	47	502	53	583	61	373	39	294	31	59	79	79	8	17	21
Tachycardia and/or HBP	561	59	395	41	587	61	369	39	257	27	65	70	112	12	20	30
Anosmia and ageusia	662	69	294	31	862	90	94	10	84	9	29	89	10	1	2	11
Cutaneous signs	745	78	211	22	805	84	151	16	85	9	40	56	66	7	9	44

HBP: high blood pressure. N: total participants (956). N1: without symptoms at D0. N2: with symptoms at D0. N3: without symptoms at D60+. N4: with symptoms at D60+. N5: with symptom at D0 and with symptom at D60+. N6: without symptom at D0 and with symptom at D60+.

a Prevalence of the self-reported symptom at D0.

b Prevalence of the self-reported symptom at D60+.

c Persistence from D0 to D60+ of the self-reported symptom.

d Incidence of the self-reported symptom at D60+.

### Cutaneous signs

The percentage of participants reporting self-reported cutaneous signs (such as itching, red patches or pseudo-chilblain lesions) decreased from 22% (211/956) at D0 to 16% (151/956) at D60+. Among the 151 participants who reported cutaneous signs at D60+, 85 (56%) reported cutaneous signs at D0 and 66 (44%) did not (Table 2). Among the variables associated with the prevalence of cutaneous signs at D60+ in the univariate analysis, those which remained significantly and independently associated with the outcome were: presence of cutaneous signs at D0 (AOR=6.42; p<0.001), female gender (AOR=2.56; p=0.021), smoking (AOR=2.34; p=0.001), and treatment(s) to manage initial COVID-19 symptoms (AOR=2.07; p=0.006) (Table 3).

**Table 3:** Univariate and multivariate logistic regression for independent factors associated with the prevalence of self-reported cutaneous signs at D60+

*Variable (Baseline, D0)*	*Presence at D60+*	*Univariable analysis (N=956)*		*Multivariable analysis (N=589)*	
	*n (%)*	*OR (95% CI)*	*p*	*AOR (95% CI)*	*p*
**Sex**					
Male (Ref.)	19 (13)	1			
Female	132 (87)	1.75 (1.07–3.00)	0.032	2.56 (1.22–6.10)	0.021
**Cutaneous signs**					
No (Ref.)	66 (44)	1			
Yes	85 (56)	6.94 (4.79–10.12)	<0.001	6.42 (3.97–10.46)	<0.001
**Treatment(s)**					
No (Ref.)	53 (36)	1			
Yes	94 (64)	1.5 (1.05–2.18)	0.028	2.07 (1.24–3.53)	0.006
**Smoking**					
No (Ref.)	97 (64)	1			
Yes	54 (36)	2.25 (1.54–3.27)	<0.001	2.34 (1.39–3.92)	0.001

AOR: adjusted odds ratio.

### Tachycardia and/or HBP symptoms

The percentage of participants reporting tachycardia and/or HBP was stable between D0 and D60+; 41% (395/956) of participants reported tachycardia and/ or HBP at D0 and 39% (369/956) at D60+. Among the 369 participants who reported tachycardia and/ or HBP at D60+, 257 (70%) reported tachycardia and/or HBP at D0, and 112 (30%) did not (Table 2). Among the variables associated with incident tachycardia and/or HBP at D60+ in the univariate analysis, attending an emergency department at D0 (AOR=2.28; p=0.001) and smoking (AOR=2.05; p=0.008) remained independently and positively associated with the outcome (Table 4).

**Table 4:** Univariate and multivariate logistic regression for independent factors associated with the incidence of self-reported tachycardia and/or HBP at D60+

*Variable (Baseline, D0)*	*Presence at D60+*	*Univariable analysis (N=540)^a^*		*Multivariable analysis (N=487)*	
	*n (%)*	*OR (95% CI)*	*p*	*AOR (95% CI)*	*p*
**Age** (years)					
20–40 (Ref.)	60 (56)	1			
40–50	37 (35)	0.63 (0.40–1.00)	0.053	0.54 (0.32–0.91)	0.021
>50	10 (9)	0.34 (0.16–0.66)	0.003	0.39 (0.17–0.8)	0.015
**Shortness of breath**					
No (Ref.)	46 (43)	1			
Yes	62 (57)	0.68 (0.44–1.05)	0.08	0.54 (0.33–0.89)	0.015
**Anosmia and ageusia**					
No (Ref.)	81 (75)	1			
Yes	27 (25)	0.63 (0.39–1.01)	0.061	0.56 (0.32–0.96)	0.039
**COVID-19 emergency**					
No (Ref.)	57 (54)	1			
Yes	49 (46)	1.85 (1.20–2.84)	0.005	2.28 (1.41–3.71)	0.001
**Smoking**					
No (Ref.)	74 (69)	1			
Yes	34 (31)	1.93 (1.20–3.08)	0.006	2.05 (1.20–3.47)	0.008

a Participants without self-reported tachycardia and/or HBP at D0. AOR: adjusted odds ratio.

## DISCUSSION

This is the first study showing that smoking is associated with the risk of post-acute COVID-19 syndrome, in particular prevalent cutaneous signs and incident self-reported tachycardia and/or HBP after 60 days or more. This study also shows that fatigue, headache, shortness of breath and chest pain were the most frequently self-reported symptoms in both periods, i.e. during the COVID-19 (D0) and more than 60 days later. Interestingly, female gender is associated with a higher risk of cutaneous signs, but smoking was a major correlate of both the prevalence of self-reported cutaneous signs at D60+ and the incidence of self-reported tachycardia and/or HBP after COVID-19.

In the literature, the relationship between smoking and COVID-19 has been controversial. A recent article addressed this issue citing studies which reported a low prevalence of smokers among subjects with positive SARS-CoV-2 biological tests compared to a reference population, but also studies which reported that smoking was associated with COVID-19 severity^17^. Two meta-analyses confirm that smoking increases the risk of negative outcomes in patients with acute COVID-19^18,19^. Following MEDLINE, EMBASE, CENTRAL, and Web of Science searches (December 2019 to June 2020), Reddy et al.^18^ identified 47 studies reporting smoking status in 32849 hospitalized COVID-19 patients, 8417 (25.6%) reported history of smoking and among them 1501 (4.6%) were current smokers. Following PubMed, EMBASE, Cochrane Library, Science Direct, and Google Scholar searches (until December 2020), Umnuaypornlert et al.^19^ identified 40 studies (19 conducted in China, and 12 in the USA) including a total of 369287 hospitalized COVID-19 patients. In the first meta-analysis^18^, both current and former smokers had an increased risk of severe COVID-19 and severe or critical COVID-19, compared with former and never smokers or never smokers, respectively. In the second meta-analysis^19^, both current and former smoking significantly increased the risk of disease severity and death. Conversely, no study obviously demonstrates that smoking increases the risk of specific clinical signs of post-acute COVID-19. In the study by Jones et al.^20^, smoking was not identified as an independent risk factor for ‘long COVID’, whereas a relationship between smoking and ‘long COVID’ was reported in the studies by Bai et al.^21^ and Hossain et al.^22^. In the study by Bai et al.^21^, active smoking was independently associated with higher risk of ‘long COVID’ syndrome, and in the study by Hossain et al.^22^, smoking was a predictor of ‘long COVID’ (p>0.001) and a predictor of longer duration of ‘long COVID’ symptoms. This is possibly due to the lack of studies investigating the link between smoking and post-acute COVID-19 syndrome and the limited sample size of the studied populations. The study by Jones et al.^20^ included 3151 patients with self-confirmed (71%), clinician-confirmed (12%) or test-confirmed (17%) COVID-19; among them, 310 patients reported having ‘long COVID’. The study by Bai et al.^21^ included 377 patients previously hospitalized for COVID-19, of which 260 had ‘long COVID’ defined by the persistence of at least one physical and/or psychological symptom. Finally, the study by Hossain et al.^22^ included 2198 patients with acute COVID-19 symptoms and a positive PCR test, with 495 reporting post-acute COVID symptoms for at least 4 weeks and 356 for at least 12 weeks. Furthermore, these studies usually focus on hospitalized patients and when conducted in the community, they are hampered by the absence of definitive diagnosis: SARS-CoV-2 infection was not established by a PCR test, especially at the beginning of the pandemic. Finally, post-acute COVID-19 includes a large range of symptoms, and whereas smoking may be associated with the risk of only some symptoms, post-COVID-19 syndrome is usually defined clinically or by the persistence of at least one symptom, and thus as a whole. To the best of our knowledge, this study showed for the first time that smoking was a major correlate of long-term self-reported cutaneous signs and occurrence of self-reported tachycardia and/or hypertension, after COVID-19. Although the underlying mechanisms are unknown, it can be hypothesized that they are similar to those usually involved in cardiovascular and skin diseases. Smoking is a well-known significant risk factor for cardiovascular diseases. It is also a contributing factor in some skin diseases and known to dysregulate wound healing^23^. Broadly, smoking is known to weaken the immune system. Finally, the findings of the present study and the literature tend to demonstrate the deleterious effect of smoking in COVID-19, irrespective of the patients (hospitalized or not) or the phase of the COVID-19 (acute or post-acute).

In the present study, the presence of self-reported cutaneous signs more than 60 days after COVID-19, was more frequent in participants who had had cutaneous signs during the acute COVID-19 phase. However, onset of cutaneous signs could be delayed, and a small proportion of participants with cutaneous signs at D60+ did not report cutaneous signs during acute COVID-19. The percentage of participants with cutaneous signs more than 60 days after the COVID-19 (16%) was close to that of the single-center cross-sectional survey by Salmon et al.6 (n=70; 14.3%), and the study by Tran et al.^24^ (n=1022; 15% dry skin/peeling, 9% skin rash, 9% discoloration/ swelling of hands and feet, for confirmed cases).

Our study also showed that women are at higher risk of cutaneous signs at D60+. This result is not surprising as female gender is currently considered a non-equivocal predicting factor for ‘long COVID’^20-22^. Moreover, it is well known that autoimmune diseases and allergies are more frequent in women than men^25,26^, and cutaneous manifestations following COVID-19 are often of a pseudo-allergic or vascular type^27^. Further studies investigating the underlying mechanisms of cutaneous manifestations following SARS-CoV-2 infection could be helpful to explain the relationship between cutaneous manifestations and female gender. Biological hormonal factors but also social behavioral factors may explain this result. The fact that participants (mainly women) were aged <50 years supports this hypothesis. Indeed, our study mainly included young women with benign forms of COVID-19, which was not surprising as the study was performed using Twitter, and as shown in a previous study, patients with prolonged forms of COVID-19 consisted mainly of young women^6^.

Overall, 95% of participants were never hospitalized. Our results therefore complement the scientific literature data. Most published studies involved patients with severe forms of the disease^28,29^. The study by Carvalho-Schneider et al.^12^ described symptoms at D60 in non-critical COVID-19 patients, but with a much smaller sample size. Their study population (n=150) was also mainly female (56%), but with a slightly older mean age than in our study (49 ± 15 years), which may be explained by the fact that patients were all medically monitored. However, in their study, only frequent symptoms were analyzed, in contrast to our study. In addition, social network data reported by people who have had COVID-19 highlighted symptoms that physicians would not have considered, but which can be disabling, such as tachycardia and/or HBP or cutaneous signs.

### Strengths and limitations

Our study has several limitations. Firstly, our sample is a convenience sample. All participants were recruited via Twitter and selection bias cannot be excluded. We analyzed self-reported data, so information bias may have occurred. Moreover, a study supervised by a physician would have probably separated HBP and tachycardia, and allowed to conclude on the impact of smoking on either HBP and/or tachycardia. This is another limitation of the present study. An additional limitation was that COVID-19 testing was not universally available during the study period; however, we selected participants with the most classical symptoms at onset. Finally, due to the study design, data were not monitored.

The first strength of the study is that it provides real-life data for a large French population during the beginning of the health crisis in France, when diagnostic tests were not available. Thus, these data are from the first infected people. In the face of new emerging SARS-CoV-2 strains, knowing the symptoms caused by the initial strain is valuable for tracking the evolution of pathogenicity and herd immunity. Secondly, the survey analyzed data from a sample of 956 participants evenly distributed throughout France. Thirdly, as previously mentioned, 95% of participants had not been hospitalized, thus providing information on non-severe COVID-19. Finally, this survey was created by a community member with symptoms at least more than 20 days after COVID-19 to share her experience with other individuals experiencing the same situation. This community-initiated survey gives an insight into the feelings and medical management of the population at the beginning of the pandemic in France. This study demonstrates that online surveys are powerful tools, easy to use, enabling rapid collection of large amounts of data. The involvement of patients in the healthcare system seems essential. A growing number of patients wish to be actors in the management of their own health and to advance medicine. In France, a community named ‘Compare, Community of Patients for Research’ has also launched a study on ‘long Covid’; patients detailed their symptoms and daily difficulties, highlighting 50 manifestations of these long forms^30^.

## CONCLUSIONS

Long-term consequences of SARS-CoV-2 infection remain a concern for the health system, and involvement of people who have had COVID-19 is helpful to quickly collect information. Though further studies are required to confirm and better understand the underlying mechanisms of the associations found, the incidence of self-reported tachycardia and/or hypertension is not negligible and suggests an interaction between COVID-19 and smoking. Reinforcing symptoms monitoring of people after acute COVID-19, mainly women and smokers, and expanding the promotion of smoking cessation strategies, are novel priorities in this COVID-19 era.

## Data Availability

The data supporting this research are available from the authors on reasonable request.
