# Supplementary material for: Smoking increases the risk of post-acute COVID-19 syndrome: Results from a French community-based survey
Source: Tob Induc Dis. 2022 Jun 17;20:59. doi: 10.18332/tid/150295 (PMC9204712; doi:10.18332/tid/150295)
Supplement: Supplementary file 1 [file TID-20-59-s1.pdf]

Supplementary Figure 1. Location of study participants.

City	N	City	N	City	N	City	N	City	N	City	N	City	N
Abbeville	1	Béziers	1	Colmar	7	Laval	1	Nancy	13	Rodez	1	Troyes	5
Agen	1	Bilieu	1	Colombes	1	Le Havre	5	Nanterre	1	Romainville	1	Valence	6
Aix-en-Provence	4	Blois	4	Compiègne	2	Le mans	6	Nantes	34	Rouen	3	Valenciennes	5
Aix-les-Bains	1	Bordeaux	17	Creil	1	Lens	1	Narbonne	3	Saint-Brieuc	1	Valréas	1
Ajaccio	3	Bourg-en-Bresse	2	Créteil	3	Lille	21	Nevers	1	Saint-Denis	2	Vannes	6
Ales	1	Bourges	1	Dieppe	1	Limoges	4	Nice	14	Saint-Die-des-Vosges	1	Vendôme	1
Ambert	1	Bourgoin-Jallieu	1	Dijon	14	Lisieux	2	Nîmes	7	Saint-Etienne	7	Verdun	2
Amiens	5	Brest	4	Douai	1	Lorient	9	Niort	1	Saint-Lô	2	Versailles	4
Angers	4	Brignoles	1	Draveil	1	Lyon	50	Noisy-le-Sec	1	Saint-Malo	1	Vesoul	1
Angoulême	2	Brive-la-Gaillarde	3	Dunkerque	3	Mâcon	2	Orléans	3	Saint-Ouen-sur-Seine	1	Vichy	2
Annecy	4	Brochant	1	Epinal	4	Mantes-la-Jolie	1	Paris	269	Saint-Quentin	1		
Antibes	2	Caen	9	Etampes	1	Marines	1	Pau	2	Sarcelles	1		
Antony	1	Calais	3	Evreux	2	Marne-la-Vallée	1	Périgueux	2	Sceaux	1		
Armentières	1	Cannes	3	Evry	3	Marseille	19	Perpignan	11	Sélestat	2		
Arras	4	Caudebec-en-Caux	1	Figeac	1	Massy	1	Pointe-à-Pitre	2	Sens	1		
Aulnay-sous-Bois	1	Cergy-Pontoise	3	Fontainebleau	1	Meaux	4	Poitiers	1	Sèvres	1		
Auxerre	3	Chalon-sur-Saône	2	Fort-de-France	2	Metz	12	Pontarlier	1	Soissons	1		
Avignon	2	Chambéry	6	Fréjus	2	Moissy-Cramayel	1	Pornichet	1	Strasbourg	27		
Bar-le-Duc	1	Chamonix	1	Grenoble	13	Montélimar	1	Puteaux	1	Tarbes	5		
Bastia	2	Chantilly	1	Guebwiller	1	Montpellier	22	Quimper	5	Thionville	2		
Bayonne	1	Chartres	2	La Rochelle	2	Montreuil	1	Rambouillet	1	Thonon-les-Bains	1		
Beauvais	2	Chatillon	1	La Roche-sur-Yon	1	Morlaix	1	Reims	19	Toulon	4		
Belfort	3	Cholet	1	Lannion	1	Mougins	1	Rennes	17	Toulouse	48		
Besançon	14	Clermont-Ferrand	6	Laon	2	Mulhouse	13	Roanne	2	Tours	7		

> 100 participants

50-100 participants

10-49 participants

5-9 participants

< 5 participants

N: number of participants.

© 2022 Barthélémy H. et al.
